# Menstrual cycle and prior sleep shape women’s responses to savory snacks during a mock night shift

**DOI:** 10.1093/sleep/zsaf362

**Published:** 2025-11-11

**Authors:** Elisa M S Meth, Diana A Nôga, Ellika Irajpour, André P Pacheco, Pei Xue, Christian Benedict

**Affiliations:** Department of Pharmaceutical Biosciences, Uppsala University, Uppsala, Sweden; Department of Pharmaceutical Biosciences, Uppsala University, Uppsala, Sweden; Department of Pharmaceutical Biosciences, Uppsala University, Uppsala, Sweden; Department of Pharmaceutical Biosciences, Uppsala University, Uppsala, Sweden; Department of Research and Innovation, Division of Mental Health and Addiction, Oslo University Hospital, Oslo, Norway; Institute of Clinical Medicine, Faculty of Medicine, University of Oslo, Oslo, Norway; Department of Pharmaceutical Biosciences, Uppsala University, Uppsala, Sweden; Department of Pharmaceutical Biosciences, Uppsala University, Uppsala, Sweden

**Keywords:** menstrual cycle, sleep, night shift, women, appetite

## Introduction

Nocturnal wakefulness during night shifts is often accompanied by late-evening eating, which has been linked to adverse body composition, impaired daytime glucose tolerance, and increased cardiovascular strain [1–4]. Food appeal during night work may be shaped by menstrual cycle–related hormonal status and total sleep time (TST) before the shift, with higher progesterone levels, typically seen during the luteal phase, and sleep durations below the recommended 7 h both known to increase food appeal and intake [5, 6]. To investigate these factors, we conducted a laboratory-based simulated night shift in reproductive-aged women, measuring sympathetic arousal with eye-tracking pupillometry and assessing palatability and wanting ratings. We hypothesized that a higher progesterone-to-estradiol (P/E) ratio and shorter prior sleep would increase both the palatability and the wanting of savory snacks during the simulated night shift.

## Materials and Methods

### Participants and study procedure

The study was conducted in accordance with the Declaration of Helsinki and was approved by the Regional Ethical Review Board in Uppsala, Sweden. Written informed consent was obtained from all participants prior to enrollment. The study was preregistered on the Open Science Framework (https://osf.io/hq2mj/) and additionally registered on ClinicalTrials.gov for transparency (NCT06683248).

The selection process is described in the Supplementary Material S1. The final sample consisted of 47 women of reproductive age. Upon arrival at the laboratory, participants received a standardized meal at 20:00 (~500 kcal; ~65 g carbohydrate, ~20 g fat, ~20 g protein) and subsequently completed an in-laboratory sleep night (lights off ~23:00; lights on ~07:00). TST was measured using a Dreem 3 headband (Beacon Biosignals, Inc., Boston, MA, USA), which has been shown to provide reliable estimates of TST when compared with consensus scoring from experienced human raters [7].

The following morning, fasting venous blood samples for estradiol (0.04–1.13 nmol/L) and progesterone (0.3–67.0 nmol/L) were collected at 07:30. Hormones were assayed using electrochemiluminescence immunoassay (Roche Cobas Pro, Roche Diagnostics) by the Clinical Laboratory at Uppsala University Hospital, Sweden, and the P/E ratio was calculated. Because neither hormone exhibits diurnal variation in women, a single morning sample provides a valid assessment of menstrual cycle status [8]. On the mock night shift day, participants were instructed to avoid any strenuous physical activity, have lunch as their last meal, and refrain from consuming caffeinated beverages. They did not eat again until dinner at 20:00, when they were provided with the same standardized meal as that served prior to the sleep-night condition. During the in-laboratory mock night shift, participants remained awake under continuous supervision and engaged in quiet activities such as board games, watching television, or reading. No additional food or beverages were permitted after dinner.

### Eye-tracking paradigm

Participants completed three eye-tracking sessions during the mock night shift (21:00, 02:00, and 04:00). In each session, they rested their heads on a headrest and fixated on a central cross displayed on a Tobii Pro Spectrum eye tracker (Tobii Technology AB, Stockholm, Sweden) at 60 Hz with binocular tracking. Stimuli were presented on a 23.8-in. monitor (1920 × 1080 resolution) against a uniform gray background (~23.24 cd/m^**2**^), with ambient lighting maintained at ~180 lux at eye level. After a 10-s baseline period, a single Pringle chip (Original flavor; ~41 per cent carbohydrate, ~54 per cent fat, ~5 per cent protein by energy) was placed in the oral cavity, and participants were instructed to keep it in their mouth without swallowing. Peak pupil dilation, an index of sympathetic arousal to a stimulus ([9]; in the present study, tasting a savory, salty crisp), was defined as the maximum pupil diameter during the subsequent 60-s exposure, expressed as the percentage increase relative to the upper bound of the 95% confidence interval from the final 5 s of the baseline period. This method accounts for baseline variability and ensures that changes reflect a genuine stimulus-evoked response. Pupil data were processed in Python following procedures adapted from PupillometryR guidelines: blinks and signal loss were excluded, pupil data within a 100-ms window surrounding each blink were discarded, extreme values exceeding three standard deviations from the median were removed, and the remaining data were smoothed with a Hann window (size 5) to reduce high-frequency noise. Finally, data from the left and right eyes were averaged to yield a single continuous measure per participant.

Following each eye-tracking session, participants rated the chip’s palatability on a Likert scale ranging from –5 (not palatable at all) to +5 (extremely palatable) and indicated their desired intake by reporting the number of chips they would choose to eat.

### Statistical analyses

Linear mixed-effects models were conducted in SPSS (version 26) using restricted maximum likelihood estimation, with subject included as a random intercept to account for repeated measures. The P/E ratio was log-transformed and then 2 was added to avoid negative values (i.e. negative values in the untransformed log corresponded to instances where estradiol exceeded progesterone in nmol/L) and treated as a continuous predictor. TST was dichotomized at 7 h (41 per cent of participants <7 h), consistent with daily sleep duration recommendations [10], to facilitate interpretation and visualization. Time point was included as a categorical covariate. A *p*-value <.05 was considered statistically significant.

## Results

**Figure 1:**
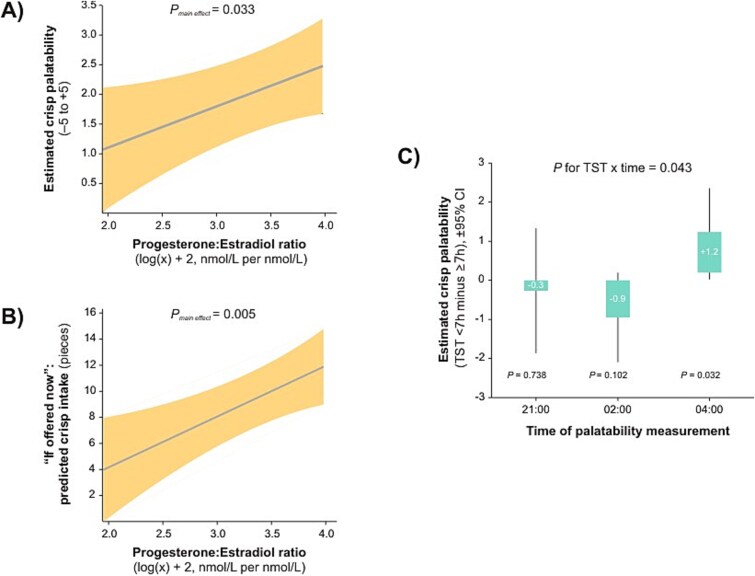
Association between the progesterone-to-estradiol ratio, total sleep time, and crisp-related outcomes. (A) Predicted crisp palatability (solid line) with 95% confidence interval (shaded area) as a function of the progesterone:estradiol ratio (log(*x*) + 2, nmol/L per nmol/L). Estimates were derived from a linear mixed-effects model including total sleep duration on the night before the mock night shift, the progesterone-to-estradiol ratio (measured on the morning of the mock night shift day), and time points (21:00, 02:00, 04:00), with subject included as a random factor. No interaction with time was observed; displayed estimates are averaged across time points. The *x*-axis is limited to the observed range of ratios. (B) Predicted hypothetical crisp selection (solid line) with 95% confidence interval (shaded area) as a function of the progesterone:estradiol ratio (log(*x*) + 2, nmol/L per nmol/L). Estimates were derived from a linear mixed-effects model including total sleep duration on the night before the mock night shift, the progesterone-to-estradiol ratio, and time points (21:00, 02:00, 04:00), with subject included as a random factor. No interaction with time was observed; displayed estimates are averaged across time points. The *x*-axis is limited to the observed range of ratios. (C) Predicted mean differences in crisp palatability between total sleep time (TST) <7 h and TST ≥7 h (bars) with 95% confidence intervals (vertical lines) at each time point. The 0 line represents the reference group (TST ≥7 h). TST was measured on the night before the mock night shift and categorized as <7 h (range: 5.8 to <7 h, *n* = 19) and 7–8.1 h (*n* = 28), consistent with the daily sleep duration recommended by the AASM. Estimates were derived from a linear mixed model including total sleep duration on the night before the mock night shift, predicted crisp palatability, and time points (21:00, 02:00, 04:00), with subject as a random factor. An interaction between TST and time was observed; therefore, displayed estimates are shown separately for each time point.

The analytic sample consisted of 47 women of reproductive age (mean ± *SD*: age, 23.9 ± 0.9 years; BMI, 21.9 ± 1.8 kg/m^2^; TST on the night preceding the mock night shift, 5.8–8.1 h). Higher log-transformed P/E ratios, indicative of a relatively higher progesterone level relative to estradiol, were associated with greater palatability ratings (*β* = 0.7 ± 0.3, *p* = .033) and higher hypothetical crisp choice (*β* = 3.9 ± 1.3, *p* = .005) (Figure 1A and B). These associations did not vary over time (interactions with time point: *p* ≥ .176). TST did not explain variability in hypothetical crisp selection across groups (≥7 h vs. <7 h: 8.2 ± 0.9 vs. 8.6 ± 1.1, *p* = .729; interaction with time point: *p* = .104). However, shorter TST was associated with higher palatability scores at 04:00, but not at other time points (interaction with time point: *p* = .043) (Figure 1C). Peak pupil dilation was not significantly associated with log-transformed P/E ratio (*β* = 1.9 ± 0.02 per cent change from pre-crisp baseline, *p* = .376; interaction *p* = .584) or TST (≥7 h vs. <7 h: 19.8 ± 1.3 per cent vs. 18.0 ± 1.5 per cent change from baseline, *p* = .336; interaction *p* = .747).

Independent of P/E ratio and TST, the linear mixed model revealed a main effect of time on hypothetical crisp selection (*p* = .014). Selection was lower at 04:00 (5.4 ± 0.9) compared with earlier sessions (02:00: 9.9 ± 1.5, *p* = .017; 21:00: 9.8 ± 1.6, *p* = .018). Palatability ratings did not differ significantly across time points (21:00: 2.4 ± 0.3; 02:00: 2.8 ± 0.3; 04:00: 1.9 ± 0.3; *p* = .167), nor did peak pupil dilation (21:00: 17.2 ± 1.4 per cent; 02:00: 20.4 ± 1.4 per cent; 04:00: 19.1 ± 1.5 per cent; *p* = .098).

## Discussion

Snacking on savory foods during night shifts may be more likely in women with higher P/E ratios, which are typically elevated in the luteal phase. This finding aligns with prior work showing that food becomes more appealing after ovulation [5]. Sleep duration also shapes daytime food choices [6]. Extending this, we found that women who slept fewer than 7 h rated crisps as more palatable toward the end of the mock night shift. Yet this heightened palatability did not translate into greater hypothetical crisp selection, which dropped to about half at 04:00 compared with earlier time points (21:00 and 02:00). Daytime sleepiness has been shown to compete with other behavioral drives [11]. Likewise, excessive fatigue at the end of the shift may suppress “wanting” for salty, crispy snacks, even when they are rated as more palatable, as seen among women who slept fewer than 7 h the previous night.

Taken together, these results suggest that both hormonal status and prior sleep shape the appeal of savory foods during night work, highlighting potential strategies such as ensuring at least 7 h of sleep before night shifts and offering healthier savory snacks, such as roasted nuts, instead of highly processed options like crisps, to take advantage of the effects of menstrual cycle–related shifts in progesterone on the liking and wanting of savory, salty foods. Future studies could include men, consider additional sensory modalities (e.g. sweet foods, which engage distinct neural pathways compared with savory, salty foods [11]), account for individual taste preferences, and track daytime food intake preceding the night shift to explore how prior eating patterns might interact with sleep and hormonal status in shaping appetite and food preferences.

## Supplementary Material

Supplement_SLEEP-2025-0908_zsaf362
