# Supplementary material for: Menstrual cycle and prior sleep shape women’s responses to savory snacks during a mock night shift
Source: Sleep. 2025 Nov 11;49(3):zsaf362. doi: 10.1093/sleep/zsaf362 (PMC13017556; doi:10.1093/sleep/zsaf362)
Supplement: Supplement_SLEEP-2025-0908_zsaf362 [file supplement_sleep-2025-0908_zsaf362.docx]

**Supplementary Information for “Menstrual Cycle and Prior Sleep Shape Women’s Responses to Savory Snacks During a Mock Night Shift”**

Elisa M. S. Meth^1^, Diana A. Nôga^1^, Ellika Irajpour^1^,

André P. Pacheco^1,2,3^, Pei Xue^1^, Christian Benedict^1*^

**^1^** Department of Pharmaceutical Biosciences, Uppsala University, Husargatan 3, Box 591, 751 24 Uppsala, Sweden.

^2^ Department of Research and Innovation, Division of Mental Health and Addiction, Oslo University Hospital, Sognsvannsveien 21, 0372 Oslo, Norway

^3^ Institute of Clinical Medicine, Faculty of Medicine, University of Oslo, Postboks 1039 Blindern, 0315 Oslo, Norway.

***Correspondence to:**

Christian Benedict, Department of Pharmaceutical Biosciences, Uppsala University, Husargatan 3, Box 591, 751 24 Uppsala, Sweden. E-mail: christian.benedict@uu.se

*Sample Selection Process*

Of 673 women who expressed interest, 288 were screened for eligibility. Exclusion criteria included age <18 or >35 years; history of physical, psychiatric, or sleep disorders; current or recent night-shift work; use of hormonal contraceptives or chronic medications; irregular menstrual cycles (outside 26–35 days); pregnancy; recent transmeridian travel; habitual sleep onset before 22:00 or after 00:00; poor sleep quality or recurrent insomnia; habitual sleep duration <7 or >9 hours; regular nicotine use; and BMI <18.5 or >25 kg/m². Screening comprised an online questionnaire followed by an in-person visit to verify eligibility and assess height and weight.

Sixty women were initially enrolled. Two withdrew before the adaptation night, leaving 58 participants who completed a laboratory-based adaptation night designed to minimize first-night effects. Participants unable to sleep during this night or with an apnea–hypopnea index ≥5 events/h were excluded. After these exclusions and additional withdrawals, 54 women completed the baseline sleep night (lights off ~23:00; lights on ~07:00) preceding the in-laboratory mock night shift.

Fasting blood samples for hormonal analysis could not be obtained from seven participants due to withdrawal of consent or insufficient sample volume. Therefore, the final analytical sample used to examine whether TST the night before a mock night shift or menstrual hormone status predicted pupil dilation or crisp ratings consisted of 47 women.
